# A case report: a heterozygous deletion (2791_2805 del) in exon 18 of the filamin C gene causing filamin C-related myofibrillar myopathies in a Chinese family

**DOI:** 10.1186/s12883-018-1078-4

**Published:** 2018-06-04

**Authors:** Jing Miao, Fei-fei Su, Xue-mei Liu, Xiao-jing Wei, Yun Yuan, Xue-fan Yu

**Affiliations:** 1 Department of Neurology and Neuroscience Center, First Affiliated Hospital of Jilin University, Changchun, 130021 Jilin, People’s Republic of China; 2 Department of Neurology, Peking University First Hospital, #8 Xishiku St, Xicheng District, Beijing, 100034 China; 3 Department of Neurology and Neuroscience Center First Affiliated Hospital of Jilin University, Changchun, 130021 Jilin, People’s Republic of China

**Keywords:** Deletion, Filamin C gene, Dominant, Myofibrillar myopathy, Case report

## Abstract

**Background:**

Filamin C-related myofibrillar myopathies (MFM) are progressive skeletal myopathies with an autosomal dominant inheritance pattern. The conditions are caused by mutations of the filamin C gene (*FLNC*) located in the chromosome 7q32-q35 region. Genetic variations in the *FLNC* gene result in various clinical phenotypes.

**Case presentation:**

We describe a 43-year-old woman who suffered filamin C-related MFM, with symptoms first presenting in the proximal muscles of the lower limbs and eventually spreading to the upper limbs and distal muscles. The patient’s serum level of creatine kinase was mildly increased. Mildy myopathic changes in the electromyographic exam and moderate lipomatous alterations in lower limb MRI were found. Histopathological examination revealed increased muscle fiber size variability, disturbances in oxidative enzyme activity, and the presence of abnormal protein aggregates and vacuoles in some muscle fibers. Ultrastructural analysis showed inclusions composed of thin filaments and interspersed granular densities. DNA sequencing analysis detected a novel 15-nucleotide deletion (c.2791_2805del, p.931_935del) in the *FLNC* gene. The patient’s father, sister, brother, three paternal aunts, one paternal uncle, and the uncle’s son also had slowly progressive muscle weakness, and thus, we detected an autosomal dominant inheritance pattern of the disorder.

**Conclusions:**

A novel heterogeneous 15-nucleotide deletion (c.2791_2805del, p.931_935del) in the Ig-like domain 7 of the *FLNC* gene was found to cause filamin C-related MFM. This deletion in the *FLNC* gene causes protein aggregation, abnormalities in muscle structure, and impairment in muscle fiber function, which leads to muscle weakness.

## Background

Filamin C-related myofibrillar myopathies (MFM) caused by mutations in the filamin C gene (*FLNC)* are autosomal dominant inherited and characterized by progressive myopathies that eventually result in wheelchair dependence. The *FLNC* gene is located in the 7q32-q35 chromosome band and includes 48 coding exons. To date, a continually increasing number of mutations in the *FLNC* gene have been reported in various populations worldwide. Among these mutations, codon 2710 is a mutational hotspot [1]. Mutations in different functional domains of filamins can cause various clinical phenotypes [2, 3]. Most patients with mutations in FLNC-Ig-like domains 7, 22, and 24 present with weakness in limb-girdle muscles, cardiomyopathy, and typical protein aggregation [1, 2, 4, 5], while those with mutations in the actin-binding domains or Ig-like domain 15 mostly present with weakness in the distal muscles and no cardiomyopathy or typical pathology [6, 7]. Here, we report a Chinese family suffering from filamin-C-related MFM caused by a novel 15-bp deletion in exon 18 of the *FLNC* gene.

## Case presentation

**Fig. 1:**
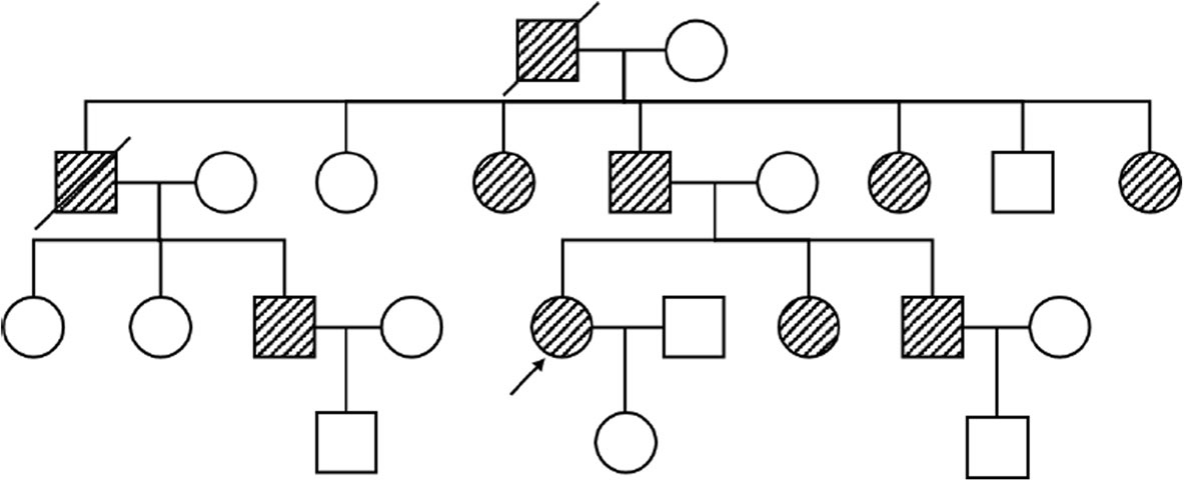
Pedigree of the patient’s family. The affected members are indicated with shading. Squares and circles represent males and females, respectively. Arrow indicates the case of the report

**Fig. 2:**
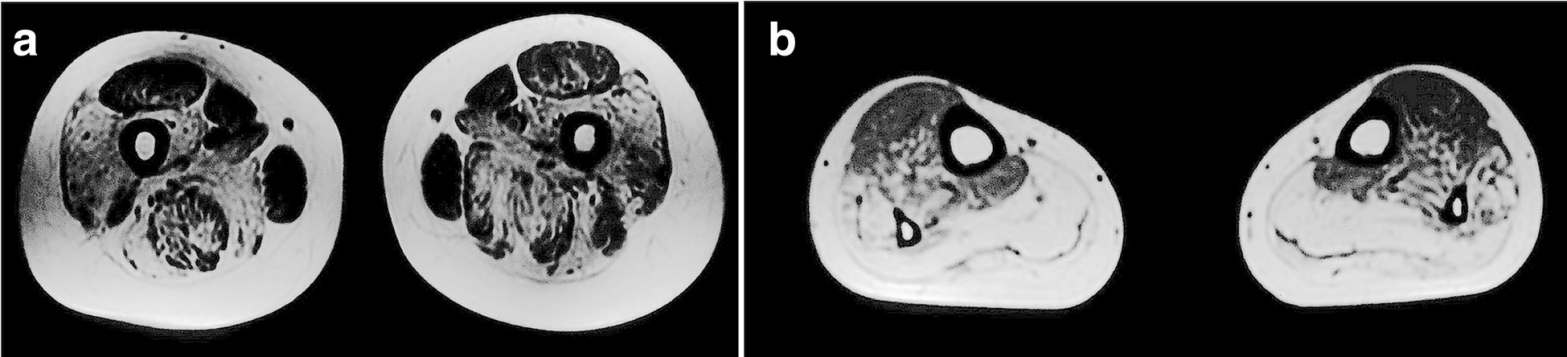
Image and transverse T1-weighted muscle MR image from the patient. MR images demonstrated a typical pattern of muscle involvement (hyperintensities reflect lipomatous alterations). On the thigh level **a** the vastus intermedius, vastus lateralis, semimembranosus, adductor magnus and long head of the biceps femoris muscles showed moderate lipomatous alterations. On the lower leg level **b** the soleus, gastrocnemius medialis, and gastrocnemius lateralis muscles show pronounced fatty changes

**Fig. 3:**
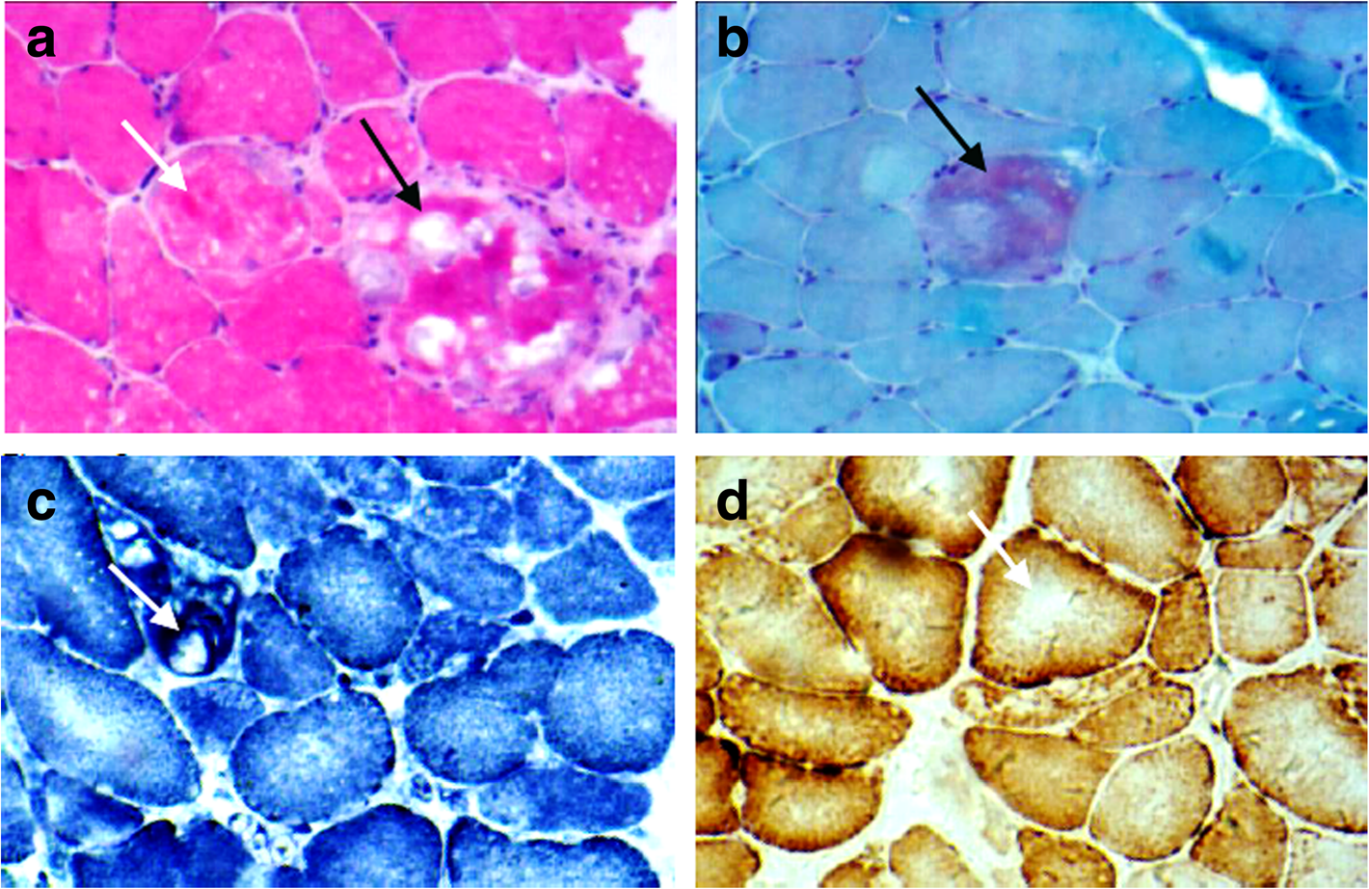
Histopathological examination of the skeletal muscles. **a** HE staining showed muscle fibers of variable sizes, vacuoles (black arrow), and abnormal material deposits (white arrow). **b** MGT staining showed abnormal material deposits in a muscle fiber (black arrow).**c** NADH-TR and (**d**) COX staining showed reduced oxidative enzyme activities in some fibers, like core-like lesions (white arrow)

**Fig. 4:**
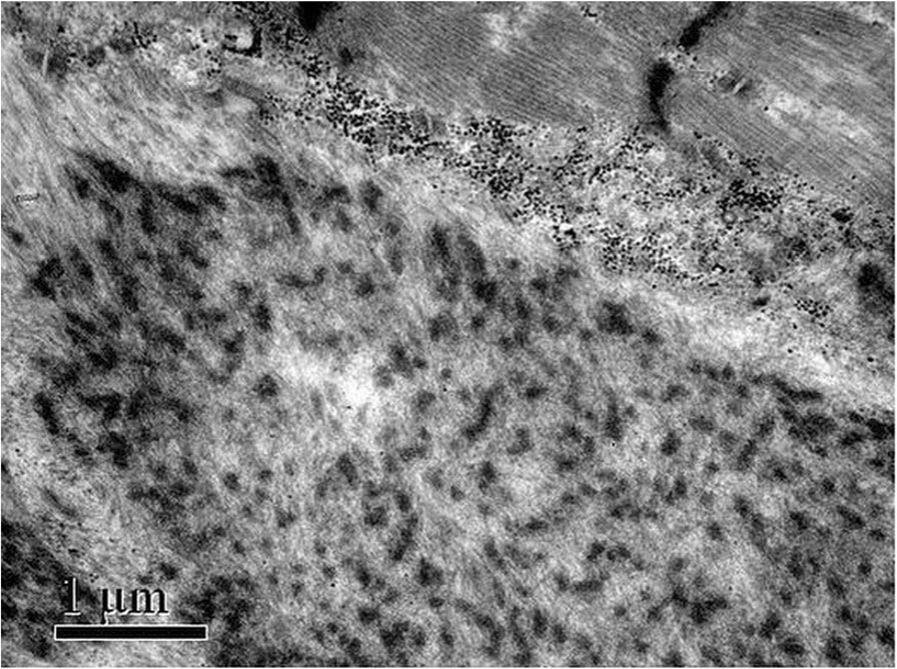
Ultrastructural examination of the skeletal muscle fibers showed deposits of dense granular material and thin filaments

**Fig. 5:**
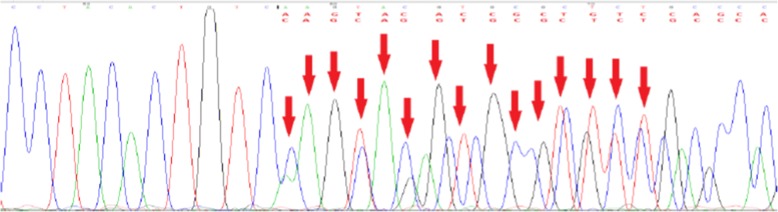
DNA sequencing analysis showed an internal 15-nucleotide deletion in exon 18 of the *FLNC* gene (red arrows)

A 43-year-old woman suffered progressively worsening proximal muscle weakness for 10 years resulting in difficulties with fast walking, climbing stairs, and rising from a chair. She had experienced normal growth and development. She reported lumbar back pain. Now, the symptom of proximal lower limb weakness had spread to her arms, causing her difficulty in washing clothes. Her father, sister, brother, three paternal aunts, one paternal uncle, and the uncle’s son also had slowly progressive proximal muscle weakness, but not as severe as hers. The pedigree of her family revealed an autosomal dominant inheritance pattern (Fig. 1). Muscle weakness was detected in the biceps and triceps muscles (MRC4+/5), wrist flexor extensor muscles (MRC 4/5), iliopsoas muscles (MRC 3/5), and quadriceps muscles (MRC 4−/5). Her distal arm strength was 4. She had a waddling gait and muscle atrophy in the quadriceps and distal lower legs. In her most recent examination, she could not walk on her toes. Deep tendon reflexes were absent. Gower’s sign was present. No cranial nerve dysfunction or sensory disturbance was noted. Her serum level of creatine kinase was 2-fold higher than the upper normal value. An electromyogram revealed mild myogenic changes. Echocardiography and electrocardiogram evaluations did not detect any cardiac abnormalities. Fatty degeneration was detected in a few lower limb muscles by magnetic resonance imaging (MRI), including the vastus intermedius, vastus lateralis, semimembranosus, adductor magnus and the long head of the biceps femoris muscles. The rectus femoris was slightly involved. No abnormalities were detected in the gracilis, sartorius, or semitendinosus muscles. At the distal leg level, the soleus, gastrocnemius medialis, and gastrocnemius lateralis showed degeneration with fatty infiltration (Fig. 2). After the patient provided written consent, a skeletal muscle biopsy was taken from the tibial anterior muscle, precooled with isopentane, and frozen in liquid nitrogen. Frozen sections of 8 μm were prepared and histopathological examination showed increased fiber size variability with atrophic and hypertrophic fibers. Muscle fiber necrosis and phagocytosis were observed. There was a mild increase in connective tissue. Non-rimmed vacuoles were commonly present. Some fibers showed structural changes with abnormal material deposits after staining with hematoxylin-eosin (HE), modified Gomori’s trichrome (MGT) (Fig. 3a and b). Oxidative enzyme activities were reduced focally in some fibers, occasionally resulting in core-like lesions after staining with succinate dehydrogenase (SDH), NADH-tetrazolium reductase (NADH-TR), and cytochrome c oxidase (COX) (Fig. 3c and d). Electron microscopy showed myofibrillar deposits of thin filaments interspersed with dense granular material between myofibrils, frequently in spheroid shape. These structures were surrounded by radially arranged thin filaments (Fig. 4). Next generation sequencing identified a 15-nucleotide deletion (c.2791_2805del, p.931_935del) in the immunoglobulin (Ig)-like domain 7 of the *FLNC* gene (Fig. 5). Genetic testing revealed that this deletion had been passed to the patient from her father and carried by her other symptomatic relatives. The patient and her relatives were administered 10 mg per day coenzyme Q10 and some vitamins for about 1 year, but their symptoms were not relieved.

## Discussions and conclusions

In the current case, a novel 15-bp deletion in the *FLNC* gene showed an autosomal dominant inheritance pattern and caused muscle weakness in all limbs, a mildly elevated serum level of creatine kinase, slight myogenic changes on EMG, variations in muscle fiber size, and the presence of abnormal protein aggregates and vacuoles in some muscle fibers. These phenotypic manifestations were also observed in the previously identified *FLNC* gene mutation-mediated MFM cases [1, 2, 4, 5].

The *FLNC* gene is located in the 7q32-q35 chromosome band and contains 48 coding exons. In general, three different domains harbored in the filamin-C protein may have different filaminS C-related myopathy phenotypes: N-terminal acting-binding domain and the frameshift deletion c.5161delG mutation in the rod domain involved with distal muscle weakness [6, 8], 24 Ig-like repeat domains, and a C-terminal dimerization domain associated with limb-girdle proximal muscle weakness [1]. In 2016, a 15-bp (c.2786_2800del) mutation in the Ig-like domain 7 of the *FLNC* gene was reported to cause limb-girdle muscular dystrophy (LGMD) [9], whereas the novel 15-bp (c.2791_2805del) mutation in the same domain in our case obviously caused the clinical phenotypes of filamin C–related MFM and displayed a typical autosomal dominant inheritance pattern. The two deletions in the same domain are very likely to result in protein aggregation in muscle fibers, causing similar clinical phenotypes [4, 5, 10]. The variation in fiber size and the presence of necrosis and fatty degeneration in muscle tissues in our case, which were also observed in cases harboring different mutations in the same Ig-like domain [4, 5], indicate that mutations in the Ig-like domain 7 of the *FLNC* gene lead to structural changes in skeletal muscles. The presence of phagocytosis, non-rimmed vacuoles, and abnormal material deposits in the muscles in our case, which is in agreement with the observations in other Ig-like domain 7 mutation-mediated filamin C–related MFM cases [4, 5] and in cultured cells transfected with expression vectors for the *FLNC* gene with a mutation in Ig-like domain 7 [10], indicates that the 15-bp deletion in the Ig-like domain 7 of the *FLNC* gene in our case may lead to protein misfolding, trigger aggregation of the mutant filamin C protein, and decrease the efficiency of both the ubiquitin–proteasome system and autophagy system to degrade protein aggregates [10–13]. These events ultimately result in disruption of myofibrils [10, 14] and muscle weakness.

In conclusion, a novel 15-nucleotide deletion (c.2791_2805del, p.931_935del) in the Ig-like domain 7 of the *FLNC* gene was found to cause filamin C–related MFM. This deletion in the *FLNC* gene causes protein aggregation, abnormalities in muscle structure, and impairment in muscle fiber function, which leads to muscle weakness.

## Abbreviations

COX: Cytochrome c oxidase (COX
HE: Hematoxylin-eosin
LGMD: Limb-girdle muscular dystrophy
MFM: Myofibrillar myopathies
MGT: Modified Gomori-trichrome
MRI: Magnetic resonance imaging;
NADH-TR: NADH-tetrazolium reductase
PAS: Periodic acid-schiff
SDH: Succinate dehydrogenase
